# Independent Association between Sleep Fragmentation and Dyslipidemia in Patients with Obstructive Sleep Apnea

**DOI:** 10.1038/srep26089

**Published:** 2016-05-17

**Authors:** Yingjun Qian, Hongliang Yi, Jianyin Zou, Lili Meng, Xulan Tang, Huaming Zhu, Dongzhen Yu, Huiqun Zhou, Kaiming Su, Jian Guan, Shankai Yin

**Affiliations:** 1 Department of Otolaryngology-Head and Neck Surgery, Shanghai Jiao Tong University Affiliated Sixth People’s Hospital, Shanghai, 200233, China; 2 Otolaryngology Institute of Shanghai Jiao Tong University, Shanghai, 200233, China

## Abstract

Obstructive sleep apnea (OSA) is independently associated with dyslipidemia. Previous studies have demonstrated that sleep fragmentation can impair lipid metabolism. The present study aimed to identify whether sleep fragmentation is independently associated with dyslipidemia, in a large-scale, clinic-based consecutive OSA sample. This cross-sectional study was conducted among 2,686 patients who underwent polysomnography (PSG) for suspicion of OSA from January 2008 to January 2013 at the sleep laboratory. Multivariate regression analyses were performed to evaluate the independent associations between the microarousal index (MAI) and lipid profiles adjusting for potential confounders, including metabolic syndrome components and nocturnal intermittent hypoxia. The adjusted odds ratios (ORs) for various types of dyslipidemia according to MAI quartiles, as determined by logistic regression were also evaluated. MAI was found positively associated with low-density lipoprotein cholesterol (LDL-c) but not with total cholesterol (TC), triglyceride (TG) or high-density lipoprotein cholesterol (HDL-c). Furthermore, the adjusted ORs (95% confidence interval) for hyper-LDL cholesterolemia increased across MAI quartiles, as follows: 1 (reference), 1.3 (1.1–1.7), 1.6 (1.2–2.0), and 1.6 (1.2–2.1) (*p* = 0.001, linear trend). Sleep fragmentation in OSA is independently associated with hyper-LDL cholesterolemia, which may predispose patients with OSA to a higher risk of cardiovascular disease.

Obstructive sleep apnea (OSA) is a highly prevalent chronic disease affecting approximately 4–14% of men and 2–5% of women with 30–70 years of age in the past few decades^1^,^2^. This disease is characterized by recurrent episodes of upper airway collapse during sleep, leading to nocturnal intermittent oxygen desaturation and sleep fragmentation. OSA is independently associated with metabolic diseases including dyslipidemia^3^,^4^,^5^,^6^; however, which pathophysiology change of OSA underlies lipid impairments remains unclear.

Previous studies have attributed the pathophysiology of lipid metabolism disorders in OSA mainly to chronic nocturnal intermittent hypoxia^7^,^8^,^9^. In particular, the oxidative desaturation index and oxy-hemoglobin saturation of <90%, surrogate markers for hypoxia were found to be independent contributors to hypercholesterolemia and hypertriglyceridemia^10^,^11^,^12^. However, sleep fragmentation may also play a key role in dyslipidemia in OSA, since the association between metabolic disorders and sleep loss, which could be caused by total sleep deprivation^13^, chronic sleep restriction^14^, or sleep fragmentation^15^, has recently received more attention. There is strong evidence that sleep deprivation is a significant risk factor for dyslipidemia^16^. Moreover, a previous study reported an association between sleep fragmentation and dyslipidemia in a population of approximately 700 OSA patients^17^. However, the included subjects in that studies were non-consecutive, which could have induced selection bias, and the confounding effect of intermittent hypoxia on the association between sleep fragmentation and dyslipidemia was not evaluated.

In addition to these previous reports on the association between sleep fragmentation and dyslipidemia^15^, studies performed in animal models showed that repeated arousal from sleep could cause impaired lipid levels^11^,^18^. Thus, it is important to examine whether sleep fragmentation itself is associated with dyslipidemia in OSA, independently of intermittent hypoxia. We conducted this large-scale, cross-sectional study to evaluate the independent association between microarousals and whole lipid profiles in a population of consecutive OSA patients. Furthermore, adjusted odds ratios (ORs) for different dyslipidemia categories in OSA patients with different levels of sleep fragmentation were calculated.

## Results

### Baseline characteristics and univariate analysis

Of the 2,686 patients, those with elevated micro arousal index (MAI) values were more likely to be male, current smokers, current alcohol consumers and to have hypertension, diabetes, and a higher body mass index (BMI) and waist circumference (WC) (Table 1). A positive dose-response relationship was observed between MAI and total cholesterol (TC), low- density liproprotein cholesterol (LDL-c), and triglyceride (TG) levels, and a negative dose-response relationship was observed between MAI and high- density liproprotein cholesterol (HDL-c) levels. The prevalence of hypercholesterolemia, low HDL cholesterolemia, hyper-LDL cholesterolemia, and hypertriglyceridemia increased with the MAI quartile from 25.78% to 35.58%, 41.13% to 49.78%, 23.4% to 42.9% and 28.91% to 54.86%, respectively (linear trends, *p* < 0.001) (Table 2).

### Sleep parameters and fasting lipid levels

**Figure 1:**
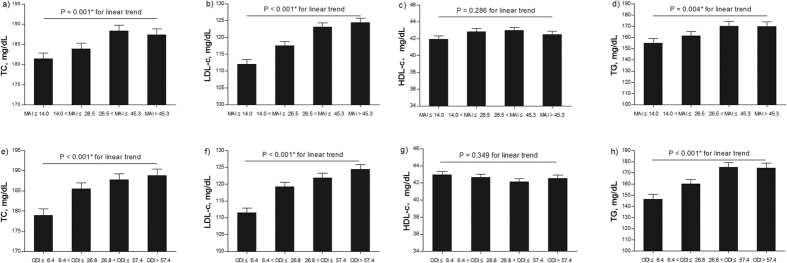
Adjusted mean values of the lipid levels in model 1. (**a**) Total cholesterol- MAI; (**b**) LDL-c-MAI; (**c**) HDL-c-MAI; (**d**) Triglyceride-MAI; (**e**) Total cholesterol- ODI; (**f**) LDL c-ODI; (**g**) HDL-c-ODI; and (**h**) Tiglyceride-ODI. The data were adjusted for age, body mass index (BMI), fasting glucose, insulin resistance, and mean arterial pressure as continuous variables, and sex, smoking status, and alcohol consumption as categorized variables. LDL- c: low-density lipoprotein cholesterol; HDL- c: high- density lipoprotein cholesterol; MAI: microarousal index; ODI: oxygen desaturation index.

According to model 1, the multiple linear regression analysis showed that the MAI was significantly associated with TC, LDL-c, and TG, but not with HDL-c (Table 3). Specifically, increasing MAI quartiles were also associated with increasing TC, LDL-c, and TG levels after adjusting for age, sex, BMI, fasting glucose level, IR, mean arterial pressure (MAP), smoking status, and alcohol consumption (linear trend, all *p* < 0.05); however, a linear trend for HDL-c among the MAI quartiles was not found (p = 0.286) (Fig. 1). The ODI in the same model was positively associated with the TC, LDL-c, and TG levels (n = 2,686; TC, β = 0.101, *p* < 0.001; LDL-c, β = 0.160, *p* < 0.001; TG, β = 0.308, *p* < 0.001) but was not associated with the HDL-c level in model 1. Linear trends for the TC, LDL-c, and TG levels among the ODI quartiles were also significant in model 1 (Fig. 1).

**Figure 2:**
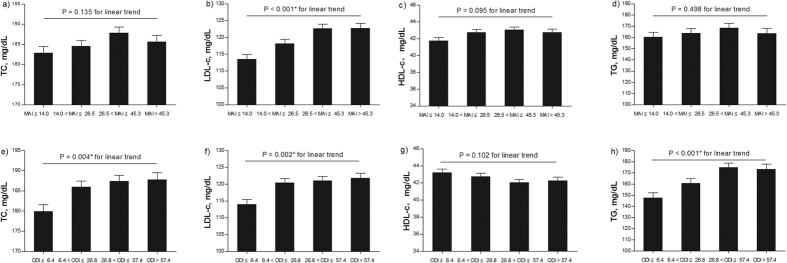
Adjusted mean values of the lipid levels in model 2. (**a**) Total cholesterol- MAI; (**b**) LDL-c-MAI; (**c**) HDL-c-MAI; (**d**) Triglyceride-MAI; (**e**) Total cholesterol- ODI; (**f**) LDL c-ODI; (**g**) HDL-c-ODI; and (**h**) Triglyceride-ODI. The data were adjusted for age, body mass index (BMI), fasting glucose, insulin resistance, and mean arterial pressure as continuous variables, and sex, smoking status, and alcohol consumption as categorized variables, with the inclusion of the ODI in (**a**–**d**) and, MAI in (**e**–**h**). LDL-c: low-density lipoprotein cholesterol; HDL-c: high-density lipoprotein cholesterol; MAI: microarousal index; ODI: oxygen desaturation index.

For model 2, when the ODI was further added as one of the covariates, the LDL-c levels remained associated with the MAI (*p* < 0.001) (Table 3). Similarly, there was a significant linear trend for the mean LDL-c value across the MAI quartiles in the fully adjusted model (*p* < 0.001) (Fig. 2), whereas the associations of the MAI with TC and TG levels were no longer significant (Table 3). The loss of the linear associations of the MAI with TC and TG levels was also seen in the adjusted mean MAI values across quartiles (*p* = 0.135 and 0.498, respectively) (Fig. 2). However, the ODI was still significantly associated with the TC and TG levels in model 2 (both *p* < 0.001) (Table 3), and the significant linear trends for the TC and TG levels among the ODI quartiles were maintained after the MAI was added into model 1 (Fig. 2), suggesting that the ODI, rather than the MAI, was responsible for the changes in the TC and TG levels.

When obesity, hypertension and diabetes were used as the categorized covariates in the linear regression analyses in models 3 and 4, the association between the MAI and the lipid levels was similar in models 1 and 3, with similar results obtained for models 2 and 4 (Table S1, Figs S1 and S2).

The hierarchical regression analysis showed that the r^2^ change in the MAI was 0.007 in model 2 and 0.008 in model 4 (both *p* < 0.001), which indicated a significant association between the MAI and the LDL-c level independent of the other covariates included in those models.

### Sleep fragmentation and dyslipidemia

We assessed the associations between the MAI and various types of dyslipidemia, including hypercholesterolemia, hyper-LDL cholesterolemia, low-HDL cholesterolemia, and hypertriglyceridemia, in the multivariate logistic models, adjusting for age, sex, BMI, fasting glucose level, IR, MAP, smoking status, and alcohol consumption in model 1, and additionally accounting for the ODI in model 2 (Table 4). In model 1, positive linear trends for the odds ratios (ORs) and 95% confidence ratio (95%CI) of all the dyslipidemia types, with the exception of low-HDL cholesterolemia, with increasing MAI quartile were determined (linear trend, all *p* < 0.05). In model 2, only the OR (95% CI) for hyper-LDL cholesterolemia remained significant in the linear trend test OR (95% CI) = 1 (reference); 1.3 (1.1–1.7), 1.6 (1.2–2.0), and 1.6 (1.2–2.1) (linear trend, *p* = 0.001) when the ODI was further taken into consideration. For models 3 and 4, the ORs and linear trends of dyslipidemia across the MAI quartiles were similar between models 1 and 3 and between models 2 and 4 (Table S2).

## Discussion

In the present large-scale cross-sectional study, we demonstrated a significant positive association between sleep fragmentation, as measured by MAI, and fasting LDL-c levels, as well as a significant increasing linear trend for adjusted mean LDL-c levels across MAI quartiles after controlling for various confounding factors, including nocturnal intermittent hypoxia. Furthermore, we demonstrated that OSA patients with higher frequencies of microarousal were at a higher risk of hyper-LDL cholesterolemia.

Since dyslipidemia is an established risk factor for morbidity and mortality of cardiovascular diseases^19^,^20^,^21^, tremendous efforts have been made to detect potential factors associated with lipid homeostasis. Specifically, the role of OSA in lipid metabolism has been assessed in multiple epidemiological studies and clinical trials. In a number of published studies, different types of dyslipidemia were not only associated with OSA^22^,^23^,^24^ but were also improved after treatment of OSA with continuous positive airway pressure^25^,^26^. However, these studies mainly investigated the impact of nocturnal intermittent hypoxia on lipid levels^7^,^9^, while the impact of sleep fragmentation, another important characteristic of OSA on lipid metabolism independently of nocturnal intermittent hypoxia, remains largely unknown. Our findings support an independent association between sleep fragmentation and hyper-LDL cholesterolemia in patients with OSA. The large sample size and the adjustment for confounding factors, including age, sex, obesity, fasting glucose, IR, blood pressure, smoking status, alcohol consumption, and nocturnal intermittent hypoxia, increased the accuracy and the reliability of our findings. The participants in our study can be assumed to demonstrate a typical pattern of OSA, as our study included a large sample of patients with a wide range of disease severities.

We found that HDL-c levels were not associated with MAI, which is in accordance with previous studies^15^,^17^,^27^. This may be due to the rather small influence of sleep fragmentation on HDL-c. In addition, although the associations of MAI with TG and TC were significant in model 1, the relationship was reduced after further consideration of the effects of nocturnal intermittent hypoxia, and a linear trend was not found across the ORs and the 95% CIs for hypertriglyceridemia or hypercholesterolemia. Previous studies have also demonstrated that a higher ODI is associated with higher TC and TG levels^12^,^17^. It can be deduced that nocturnal intermittent hypoxia may explain the increase in TC and TG levels more so than sleep fragmentation.

We postulated that sleep fragmentation plays an important role in lipid homeostasis in addition to nocturnal intermittent hypoxia in OSA patients for a number of reasons. Firstly, more fragmented sleep was reported to be associated with higher TC and LDL-c levels, as well as heart rate, blood pressure, and cortisol in a population of 24 non-OSA subjects^15^. Secondly, sleep fragmentation is a stressor that induces activation of the hypothalamic-pituitary-adrenal axis, thus causing the elevation of hormones such as adrenocorticotropic hormone and cortisol^28^. These hormones play a role in lipolysis, which might affect lipid levels^29^. Thirdly, sleep fragmentation has been found to cause systemic inflammation^30^,^31^, which can result in changes in lipid metabolism^32^. Lastly, sleep fragmentation increases energy demands, food intake, and the risk of obesity^30^,^33^. Although our observed results are independent of obesity, sleep fragmentation-induced obesity may contribute to dyslipidemia risk.

Our results are of clinical importance. Serum LDL-c is a major cause of atherosclerosis^34^, and lowering of LDL-c levels was suggested as the first-line and primary target for coronary heart disease risk-reducing therapy by NCEPIII^35^. Hyper LDL-cholesterolemia was found to be associated with more fragmented sleep independent of ODI in OSA patients in our study. Altogether, these findings suggest that dyslipidemia as an early sign of atherosclerosis should be monitored not only in OSA patients with nocturnal intermittent hypoxia, but also in patients with more fragmented sleep. Moreover, the association between sleep fragmentation and hyper LDL cholesterolemia may also help in promoting treatment of OSA, aiming to control lipid impairments.

We acknowledge a number of limitations of this study. First, the cross-sectional design of this study does not allow for conclusions to be drawn regarding a causal relationship between sleep fragmentation and lipid impairments. Experimental evidence from cohort studies and randomized controlled trials are warranted to determine a robust causal link. Second, obesity, diabetes, and hypertension are associated with dyslipidemia independently of OSA. Although these factors were adjusted in the regression analyses between the MAI and lipids, an interpretation of the complex associations between sleep fragmentation, diabetes, hypertension, and dyslipidemia in patients with OSA was nonetheless difficult. Third, residual confounders besides the included adjustments, such as diet and physical activity, could influence serum lipids, although we enrolled only residents of east China with roughly analogous lifestyles.

## Conclusion

Sleep fragmentation is independently associated with hyper-LDL cholesterolemia in OSA patients. It is warranted to investigate the causal relationship between sleep fragmentation and dyslipidemia, as well as the underlying mechanisms of this association further in prospective cohort studies.

## Methods

### Study population

Between January 2008 and January 2013, 3,224 consecutive adults (age ≥ 18 years) were referred to the sleep laboratory at the Shanghai Jiao Tong University Affiliated Sixth People’s Hospital for suspected OSA. Among these, 538 participants were excluded for the following reasons: 1) previous treatment for OSA (n = 106), 2) treatment with lipid-lowing medications before the study (n = 92), and 3) missing data (n = 340). Finally, 2,686 patients were included in this study. The study was approved by the Internal Review Board of the Institutional Ethics Committee of Shanghai Jiao Tong University Affiliated Sixth Hospital, and was conducted in accordance with the Declaration of Helsinki. Written informed consent was obtained from all participants.

### Sleep parameters

To obtain objective sleep parameters, overnight polysomnography (PSG, Alice 4 or 5: Respironics Inc., Pittsburgh, USA) was performed under attended conditions in the laboratory. The following channels were employed: bilateral central referential electroencephalogram (EEG) channels (C3-M2 and C4-M1), bilateral electroculograms (EOG), a bipolar chin electromyogram (EMG), airflow (using both nasal-oral thermocouple and nasal pressure cannula), finger pulse oximetry, chest and abdominal wall motion (piezo electrodes), electrocardiogram, and body position. Sleep stages, respiratory events, and microarousals were scored automatically using a computer software and were subsequently checked manually by a skilled technician, following the American Academic Sleep Medicine (AASM) criteria^36^. Apnea was defined as cessation of airflow for ≥10 s. Hypopnea was defined as a ≥50% reduction in airflow accompanied by a decrease in oxyhemoglobin saturation of ≥3%. A microarousal was defined as an abrupt shift in the EEG frequency, including alpha, theta and/or frequencies >16 Hz (but not spindles) that lasted at least 3 s, with at least 10 s of stable sleep preceding the change. In addition, scoring of arousal during rapid eye movement requires a concurrent increase in submental EMG lasting at least 1 s. The apnea-hypopnea index (AHI), the microarousal index (MAI), and the 3% oxygen desaturation index (ODI) were defined by the number of events per hour of recording, and the percentage of time spent at SaO_2_ < 90% (CT90) was also calculated.

### Lipid levels

For each participant, a fasting blood sample was collected from the antecubital vein the morning after PSG evaluation. The following lipid parameters were tested in the hospital laboratory using routine procedures: total cholesterol (TC), triglycerides (TG), high-density lipoprotein cholesterol (HDL-c), and low-density lipoprotein cholesterol (LDL-c). According to the US National Cholesterol Education Program Adult Treatment Panel III (NCEPIII) diagnostic criteria, dyslipidemia in terms of TC, LDL-c, HDL-c and TG were defined as TC levels >200 mg/dL, LDL-c levels >130 mg/dL, HDL-c levels < 39.8 mg/dL, and TG levels >150 mg/dL, separately^35^.

### Covariates

Anthropometric variables including weight, height and waist circumference (WC) were measured with the participant in light weight clothing and bare feet using standard methods. Body mass index (BMI) was calculated as weight in kilograms divided by height in meters squared. Waist circumference (WC) was measured horizontally, midway between the lowest rib margin and the iliac crest. Obesity was defined as a BMI ≥ 28 kg/m^2^ and abdominal obesity as a WC ≥ 85 cm for males and ≥80 cm for females based on the recommendations of the Working Group on Obesity in China^37^.

Smoking status and alcohol consumption were ascertained by means of a self- reported questionnaire completed on the night of each patient’s in-laboratory PSG recording. Smoking status was classified as current or non-current smoker. Patients were also stratified according to their reported alcohol consumption into 2 groups: current or non-current drinker. The specific questions asked in the questionnaire and the cut-off values are provided in the Supplementary Materials.

Daytime blood pressure (BP) was measured after at least 5 min of rest in a sitting position using a mercury sphygmomanometer, following the American Society of Hypertension Guidelines, and the mean of three measurements was recorded. Fasting serum glucose levels were measured in the hospital laboratory using an auto analyzer (H-7600; Hitachi, Tokyo, Japan). An immunoradiological method was used to measure the fasting serum insulin level. Mean arterial pressure was calculated as (systolic BP+ 2* diastolic BP)/3. Insulin resistance was estimated using the previously described homeostasis model of assessment (HOMA) method^38^: fasting serum insulin (μU/mL) × fasting plasma glucose (mmol/L)/22.5. Each patient also completed a survey on his or her medical history. Hypertension was defined as a systolic BP ≥ 140 mmHg, a diastolic BP ≥ 90 mmHg, or current use of antihypertensive medication. Diabetes was defined as a fasting blood glucose level >126 mg/dL, a glycated hemoglobin level ≥ 6.5%, or treatment for diabetes^35^.

### Statistical analysis

Descriptive statistics of the study population are displayed according to MAI quartiles using the mean and standard deviation (SD) for continuous variables and percentages for categorical data. *P*-values for linear trends across the four MAI groups were calculated using the polynomial linear trend test for continuous variables and using the linear-by-linear association test for dichotomous variables.

To understand the relationship between sleep fragmentation and lipid profiles including TC, LDL-c, HDL-c, and TG, stepwise multiple regression analyses were performed. Fasting lipid levels were used as dependent variables, while MAI was used as a predictor. All the regression analyses were adjusted for potential confounding factors in four models.

Dichotomous logistic regressions were used to model the associations between MAI quartiles and the ORs (95% confidence intervals, CIs) for hypercholesterolemia, hyper-LDL cholesterolemia, low HDL cholesterolemia, and hypertriglyceridemia, after adjusting for confounding factors in models 1–4. The *p*-value for linear trends was determined by examining the median MAI value for each quartile and assessing the overall F test for the median MAI variable.

The statistical analysis was preceded by the use of collinearity diagnostics to eliminate possible multicollinearity among variables. The 2 steps of the collinearity analyses were: (1) a preliminary analysis using Spearman’s correlation; and (2) collinearity diagnostics to determine the selected covariates in the multivariate regression analyses. Based on the collinearity diagnosis, in model 1 we adjusted for age, BMI, fasting glucose level, HOMA- IR, and mean arterial pressure as continuous variables, and sex, smoking status, alcohol consumption as dichotomous variables. As nocturnal intermittent hypoxia was considered a potential confounder for the association between the MAI and outcomes in patients with OSA, we also accounted for the oxygen desaturation index (ODI) in the second multivariate model. Model 3 included the following covariates: age as a continuous variable; and sex, obesity, abdominal obesity, hypertension, diabetes, smoking status, and alcohol consumption as dichotomous variables. Model 4 further added the ODI to model 3. For detailed results of the collinearity diagnoses, see Supplementary Tables 3–12.

Hierarchical regression was used to test the independent influence of the MAI in models 2 and 4. The method analyzes the effect of a predictor variable after controlling for other variables, achieved by calculating the change in the r^2^ at each step of the analysis.

Of note, ODI was examined as both a classified variable according to its quartiles as well as a continuous one. These yielded similar conclusions, and we presented only the results using the continuous variables in linear regression analyses and the quartile variables in logistic regression analyses for simplicity. All statistical analyses were conducted using SPSS software (ver. 20.0; SPSS Inc., Chicago, IL, USA). A bilateral *p*-value < 0.05 was considered statistically significant^37^,^38^.

## Additional Information

**How to cite this article**: Qian, Y. *et al*. Independent Association between Sleep Fragmentation and Dyslipidemia in Patients with Obstructive Sleep Apnea. *Sci. Rep.* **6**, 26089; doi: 10.1038/srep26089 (2016).

## Supplementary Material

Supplementary Information

## Figures and Tables

**Table 1:** Characteristics of the study participants according to MAI quartiles.

Characteristic	MAI ≤ 14.0	14.0 < MAI ≤ 26.5	26.5 < MAI ≤ 45.3	MAI > 45.3	*P*-value for trend^a^
No. patients	671	673	673	669	–
Demographics					
Age, y	42.92 ± 12.32	42.85 ± 12.02	43.54 ± 12	42.84 ± 10.95	0.054
Male	65.28	78.6	86.78	90.88	<0.001
BMI, kg/m^2^	25.11 ± 3.66	26 ± 3.54	26.47 ± 3.48	27.9 ± 3.74	<0.001
Abdominal obesity, %	75.86	86.18	90.49	95.96	<0.001
Medical history					
Hypertension, %	21.61	24.37	32.69	37.67	<0.001
Diabetes, %	6.11	7.43	7.58	11.51	<0.001
Current smokers, %	20.57	29.12	33.14	33.33	<0.001
Current alcohol consumers, %	4.02	6.24	8.77	8.97	<0.001
Sleep parameters					
Apnea hypopnea index	13.25 ± 18.56	23.44 ± 22.41	34.92 ± 22.93	57.23 ± 25.07	<0.001
Oxygen desaturation index	14.13 ± 20.46	24 ± 23.85	36.97 ± 23.85	59.53 ± 27.4	<0.001

Data are presented as means ± SD or percentages. BMI, body mass index; MAI, microarousal index.

^a^Tested using the polynomial linear trend test for continuous variables and the linear-by-linear association test for dichotomous variables.

**Table 2:** Lipid profiles and percentage of dyslipidemia according to MAI quartiles.

Characteristic	MAI ≤ 14.1	14.1 < MAI ≤ 26.5	26.5 < MAI ≤ 45.3	MAI > 45.3	*P-*value for trend^a^
No. patients	671	673	673	669	–
Biochemistry assays					
Total cholesterol, mg/dL	178.95 ± 37.17	183.06 ± 36.37	188.79 ± 38.04	190.36 ± 38	<0.001
HDL-c, mg/dL	43.57 ± 9.86	43.12 ± 10.16	42.62 ± 10.29	41.01 ± 8.82	<0.001
LDL-c, mg/dL	109.86 ± 31.88	116.81 ± 31.42	123.63 ± 33.58	126.93 ± 33.7	<0.001
Triglycerides, mg/dL	137.6 ± 101.73	156.65 ± 95.42	173.5 ± 117.52	188.92 ± 109.74	<0.001
Dyslipidemia					
Hypercholesterolemia, %	25.78	29.87	35.81	35.58	<0.001
Hyper LDL cholesterolemia, %	23.4	31.95	38.19	42.9	<0.001
Low HDL cholesterolemia, %	41.13	40.56	44.87	49.78	<0.001
Hypertriglyceridemia, %	28.91	41.9	45.47	54.86	<0.001

Data are presented as mean ± SD or %. HDL-c, high-density lipoprotein cholesterol; LDL-c, low-density lipoprotein cholesterol; MAI, microarousal index.

^a^ested using the polynomial linear trend test for continuous variables and the linear-by-linear association test for dichotomous variables.

**Table 3:** Stepwise multiple linear regression for fasting blood lipids in models 1 and 2.

Variable	Reference	TC, mg/dL	LDL-c, mg/dL	HDL-c, mg/dL	TG, mg/dL
Model 1					
Age		0.292(0.061)^a^	0.134(0.053)^c^	0.039(0.015)^c^	–
Sex	Male	–	–	5.769(0.481)^a^	−27.468(5.266)^a^
BMI		0.743(0.208)^a^	0.615(0.183)^b^	−0.435(0.056)^a^	3.439(0.612)^a^
Fasting glucose		0.229(0.041)^a^	0.135(0.036)^a^	–	0.638(0.123)^a^
IR		–	–	−0.355(0.088)^a^	7.057(1.091)^a^
MAP		0.214(0.067)^b^	0.234(0.059)^a^	0.042(0.017)^c^	–
Smoking status	non- current smoker	–	–	−1.704(0.416)^a^	10.115(4.525)^c^
Alcohol consumption	non- current drinker	–	–	4.911(0.709)^a^	–
MAI		0.101(0.032)^b^	0.196(0.028)^a^	–	0.254(0.088)^b^
Model 2					
Age, y		0.284(0.061)^a^	0.13(0.053)^c^	0.039(0.015)^c^	–
Sex	Male	–	–	5.769(0.481)^a^	−27.248(5.24)^a^
BMI		0.541(0.225)^c^	0.44(0.198)^c^	−0.435(0.056)^a^	2.796(0.647)^a^
Fasting glucose		0.225(0.041)^a^	0.129(0.036)^a^	–	0.63(0.123)^a^
IR		–	–	−0.355(0.088)^a^	6.728(1.097)^a^
MAP		0.204(0.067)^b^	0.221(0.059)^a^	0.042(0.017)^c^	–
Smoking status	non- current smoker	–	–	−1.704(0.416)^a^	9.915(4.519)^c^
Alcohol consumption	non- current drinker	–	–	4.911(0.709)^a^	–
ODI		0.101(0.028)^a^	0.068(0.03)^c^	–	0.308(0.079)^a^
MAI		–	0.156(0.033)^a^	–	–

Data are presented as β (SE [β]). Model 1 adjusted for age, body mass index (BMI), fasting glucose, insulin resistance, and mean arterial pressure as continuous variables, and sex, smoking status, and alcohol consumption as categorized variables, and plus ODI in model 2.

TC, total cholesterol; TG, triglyceride; HDL-c, high-density lipoprotein cholesterol; LDL-c, low-density lipoprotein cholesterol; BMI, body mass index; IR, insulin resistance; MAP, mean artierial pressure; MAI, microarousal index; ODI, oxygen desaturation index.

^a^p < 0.001, ^b^p < 0.01, ^c^p < 0.05.

**Table 4:** Adjusted odds ratios for dyslipidemia according to MAI categories in models 1 and 2.

	Hypercholesterolemia	Hyper LDL cholesterolemia	Low HDL cholesterolemia	Hypertriglyceridemia
Adjusted OR (95% CI) in model 1				
MAI** ≤ **14.1	1	1	1	1
14.1 < MAI** ≤ **26.5	1.162(0.91,1.485)	1.429(1.117,1.827)	0.792(0.629,0.996)	1.469(1.156,1.865)
26.5 < MAI** ≤ **45.3	1.46(1.144,1.864)	1.762(1.379,2.252)	0.872(0.692,1.1)	1.442(1.133,1.836)
MAI > 45.3	1.318(1.022,1.699)	1.922(1.494,2.474)	0.874(0.687,1.112)	1.585(1.236,2.034)
*P*-value for linear trend	0.012^*^	<0.001^*^	0.458	<0.001^*^
Adjusted OR (95% CI) in model 2				
MAI** ≤ **14.1	1	1	1	1
14.1 < MAI** ≤ **26.5	1.118(0.871,1.433)	1.349(1.051,1.731)	0.772(0.611,0.976)	1.355(1.062,1.728)
26.5 < MAI** ≤ **45.3	1.33(1.025,1.725)	1.555(1.198,2.018)	0.818(0.639,1.048)	1.213(0.938,1.568)
MAI > 45.3	1.095(0.822,1.457)	1.589(1.198,2.106)	0.838(0.639,1.099)	1.241(0.937,1.644)
*P*-value for linear trend	0.349	0.001^*^	0.293	0.267

ORs were adjusted for age, gender, BMI, fasting glucose level, insulin resistance, mean arterial pressure, smoking status and alcohol consumption in model 1, and plus oxygen desaturation index in model 2.

LDL, low-density lipoprotein; HDL, high-density lipoprotein; MAI, microarousal index.

*P*-values for linear trends were determined by examining the median MAI value for each quartile and assessing the overall F test for the median MAI variable. *P < 0.05.
