# Supplementary material for: Independent Association between Sleep Fragmentation and Dyslipidemia in Patients with Obstructive Sleep Apnea
Source: Sci Rep. 2016 May 17;6:26089. doi: 10.1038/srep26089 (PMC4869120; doi:10.1038/srep26089)
Supplement: Supplementary Information [file srep26089-s1.doc]

**Independent Association between Sleep Fragmentation and Dyslipidemia in Patients with Obstructive Sleep Apnea**

Yingjun Qian1, 2, Hongliang Yi1, 2, Jianyin Zou1, 2, Lili Meng1, 2, Xulan Tang1, 2, Huaming Zhu1, 2, Dongzhen Yu1, 2, Huiqun Zhou1, 2, Kaiming Su1, 2, Jian Guan*1,2, Shankai Yin*1, 2

1Department of Otolaryngology-Head and Neck Surgery, Shanghai Jiao Tong University Affiliated Sixth People’s Hospital, Shanghai, 200233, China.

2Otolaryngology Institute of Shanghai Jiao Tong University, Shanghai, 200233, China.

* These authors contributed equally to this work.

***Corresponding to:** Prof. Shankai Yin (email: skyin@sjtu.edu.cn) and Dr. Jian Guan (email: guanjian0606@sina.com).

**Supplementary materials and data**

**Methods**

**Questionnaires and cut- offs on smoking status and alcohol consumption**

The question on smoking habits was: “Do you smoke?” The choices were: “(i) Yes, I have smoked cigarettes per week for years/months; (ii) No, but I used to smoke cigarettes per week for years/months, and have given up smoking for months; and (iii) I never smoked regularly.” Smoking status was classified as current or non-current smoker. Current smokers were individuals who had smoked at least 1 cigarette in the previous 12 months, and included those who had quit within the past year. Non-current smokers were those who had quit more than a year earlier or had never used tobacco products regularly.

Alcohol consumption was ascertained using the questions:“(a) Please choose the frequency of your alcohol consumption: (i) never; (ii) less than once per month; (iii) 1–3 times per month; (iv) 1–2 times per week; (v) 3–4 times per week; (vi) 5–6 times per week; (vii) once per day; (viii) more than once per day ( times per day). (b) Please describe the amount and kinds of drinks each time: liquor mL, wine mL, beer mL, others .”Alcohol consumption was classified as current or non-current drinker. Current drinkers were those who had consumed any amount of alcohol during the past year. Non-current drinkers were those who had stopped consuming alcohol for at least 12 months or those who had never consumed alcohol during their lifetime.

**Results**

**Collinearity analyses results descriptions**

The results of the 2 steps of the collinearity analyses are shown in the Supplementary Tables: (1) a preliminary analysis using Spearman’s correlation in Tables S3 and S8; and (2) collinearity diagnostics to determine the selected covariates in the multivariate linear regression analyses in Tables S5–S7 and S9–S12.

Based on the inner correlations among the metabolic variables and the collinearity analyses results for models 1 and 2, when obesity, diabetes, and hypertension were considered as continuous variables in the multiple regression models, only body mass index(BMI) (representing BMI and waist circumference), fasting glucose level and HOMA-IR (representing fasting glucose level, HOMA-IR, and fasting insulin level), and mean arterial pressure (representing systolic blood pressure and mean arterial pressure) were included in the subsequent analyses. For models 3 and 4, when obesity, diabetes, and hypertension were considered as categorized covariates, obesity (BMI < 28 kg/m^2^ or ≥ 28 kg/m^2^), abdominal obesity (yes or no), prevalence of diabetes, and prevalence of hypertension were included in the subsequent analyses.

**Supplementary figure legends(sFigure 1, sFigure 2)**

**sFigure 1．**Adjusted mean values of the lipid levels in model 3. (a) Total cholesterol- MAI; (b) LDL-c-MAI; (c) HDL-c-MAI; (d) Triglyceride-MAI; (e) Total cholesterol- ODI; (f) LDL-c-ODI; (g) HDL-c-ODI; and (h) Triglyceride-ODI. Data were adjusted for age, sex, obesity, abdominal obesity, smoking status, alcohol consumption, hypertension, and diabetes. LDL-c: low-density lipoprotein cholesterol; HDL-c: high-density lipoprotein cholesterol; MAI: microarousal index; ODI: oxygen desaturation index.

**sFigure 2．**Adjusted mean values of the lipid levels in model 4. (a) Total cholesterol- MAI; (b) LDL-c-MAI; (c) HDL-c-MAI; (d) Triglyceride-MAI; (e) Total cholesterol- ODI; (f) LDL-c-ODI; (g) HDL-c-ODI; and (h) Triglyceride-ODI. Data were adjusted for age, sex, obesity, abdominal obesity, smoking status, alcohol consumption, hypertension, and diabetes, with the inclusion of the ODI in (a)–(d) and, MAI in (e)–(h). LDL-c: low-density lipoprotein cholesterol; HDL-c: high-density lipoprotein cholesterol; MAI: microarousal index; ODI: oxygen desaturation index.

**sTable (1-12)**

**sTable 1.** Stepwise multiple linear regression for fasting blood lipids in models 3 and 4.

| Variable | Reference | TC, mg/dL | LDL-c, mg/dL | HDL-c, mg/dL | TG, mg/dL |
| --- | --- | --- | --- | --- | --- |
| Model 3 |  |  |  |  |  |
| Age | … | 0.271(0.063)^a^ | - | 0.05(0.016)^b^ | - |
| Sex | Male | - | - | 5.612(0.481)^a^ | -30.528(5.124)^a^ |
| BMI | < 28 kg/m² | - | - | -2.01(0.407)^a^ | 22.062(4.578)^a^ |
| Abdominal obesity | None | 14.515(2.195)^a^ | 12.752(1.918)^a^ | -5.014(0.558)^a^ | 57.225(6.228)^a^ |
| Hypertension | None | 4.123(1.662)^c^ | 5.835(1.399)^a^ | 1.003(0.419)^c^ | 10.064(4.505)^c^ |
| Diabetes | None | - | - | -1.342(0.66)^c^ | 33.22(7.323)^a^ |
| Smoking status | Non- current smoker | - | - | -1.785(0.416)^a^ | - |
| Alcohol consumption | Non- current drinker | - | - | 5.034(0.709)^a^ | - |
| MAI | … | 0.134(0.031)^a^ | 0.212(0.027)^a^ | - | 0.388(0.088)^a^ |
| Model 4 |  |  |  |  |  |
| Age | … | 0.307(0.06)^a^ | - | 0.046(0.016)^b^ | - |
| Sex | Male | - | - | 5.464(0.486)^a^ | -26.256(5.309)^a^ |
| BMI | < 28 kg/m² | - | - | -1.78(0.427)^a^ | 16.457(4.733)^b^ |
| Abdominal obesity | None | 13.13(2.251)^a^ | 11.463(1.968)^a^ | -4.78(0.569)^a^ | 53.115(6.288)^a^ |
| Hypertension | None | - | 5.105(1.421)^a^ | 1.085(0.423)^c^ | - |
| Diabetes | None | - | - | - | 33.919(7.252)^a^ |
| Smoking status | Non- current smoker | - | - | -1.768(0.416)^a^ | 9.05(4.547)^c^ |
| Alcohol consumption | Non- current drinker | - | - | 5.023(0.708)^a^ | - |
| ODI | … | 0.144(0.025)^a^ | 0.079(0.028)^b^ | -0.016(0.007)^c^ | 0.478(0.076)^a^ |
| MAI | … | - | 0.159(0.033)^a^ | - | - |

Data are presented as β (SE [β]). Model 3 adjusted for age as a continuous variable and sex, obesity, abdominal obesity, diabetes, hypertension, smoking status, and alcohol consumption as categorized variables, and plus ODI in model 4.

TC, total cholesterol; TG, triglyceride; HDL-c, high-density lipoprotein cholesterol; LDL-c, low-density lipoprotein cholesterol; BMI, body mass index; MAI, microarousal index; ODI, oxygen desaturation index.

a p< 0.001, b p< 0.01, c p< 0.05.

|  | Hypercholesterolemia | Hyper LDL cholesterolemia | Low HDL cholesterolemia | Hypertriglyceridemia |
| --- | --- | --- | --- | --- |
| Adjusted OR (95% CI) in model 3 | | |  |  |
| MAI ≤ 14.1 | 1 | 1 | 1 | 1 |
| 14.1 < MAI ≤ 26.5 | 1.2 (0.9,1.5) | 1.4 (1.1,1.8) | 0.7 (0.6,0.9) | 1.5 (1.1,1.9) |
| 26.5 < MAI ≤ 45.3 | 1.5 (1.2,1.9) | 1.8 (1.4,2.3) | 0.8 (0.6,1.0) | 1.5 (1.2,1.9) |
| MAI > 45.3 | 1.4 (1.1,1.8) | 2.1 (1.6,2.7) | 0.9 (0.7,1.1) | 1.8 (1.4,2.3) |
| *P*-value for linear trend | < 0.001^*^ | < 0.001^*^ | 0.475 | < 0.001^*^ |
| Adjusted OR (95% CI) in model 4 | | |  |  |
| MAI ≤ 14.1 | 1 | 1 | 1 | 1 |
| 14.1 < MAI ≤ 26.5 | 1.1 (0.9,1.4) | 1.4 (1.1,1.7) | 0.7 (0.6,0.9) | 1.3 (1.1,1.7) |
| 26.5 < MAI ≤ 45.3 | 1.3 (1.0,1.7) | 1.6 (1.2,2.0) | 0.8 (0.6,1.0) | 1.2 (0.9,1.6) |
| MAI > 45.3 | 1.1 (0.8,1.5) | 1.6 (1.2,2.2) | 0.8 (0.6,1.1) | 1.3 (1.0,1.7) |
| *P*-value for linear trend | 0.082 | < 0.001^*^ | 0.077 | 0.257 |

**sTable 2.** Adjusted odds ratios for dyslipidemia according to MAI categories in models 3 and 4.

ORs were adjusted for age, sex, obesity, abdominal obesity, smoking status, alcohol consumption, hypertension, and diabetes in model 3, and plus oxygen desaturation index in model 4.

LDL, low-density lipoprotein; HDL, high-density lipoprotein; MAI, microarousal index.

*P*-values for linear trends were determined by examining the median MAI value for each quartile and assessing the overall F test for the median MAI variable. **P*<0.05.

**sTable 3.** The results of Spearman’s correlations (for selection of covariates in model 1 and model 2).


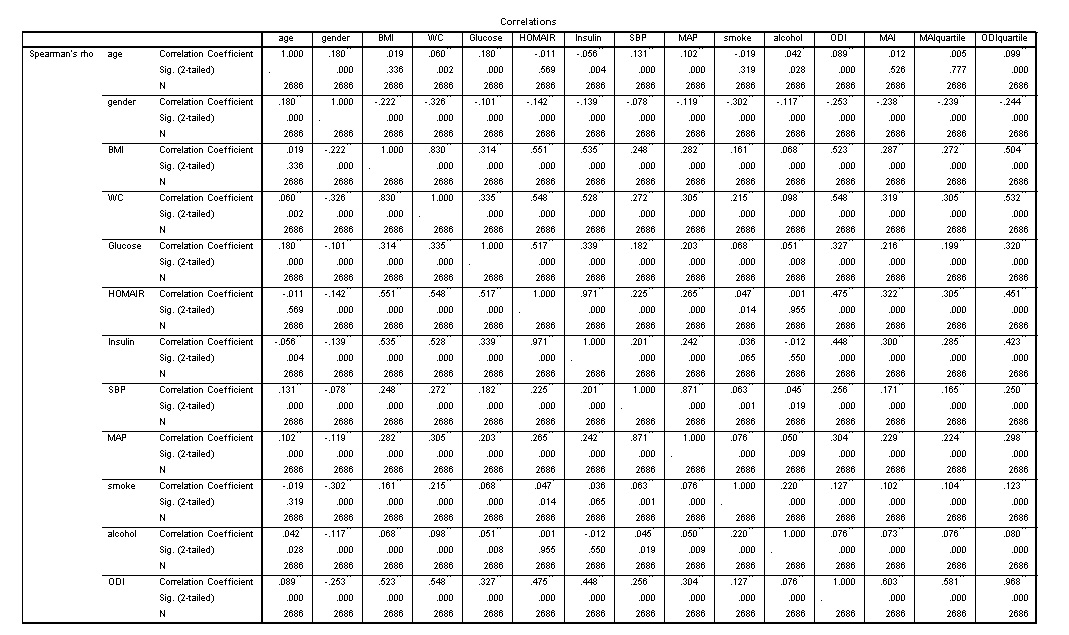


**sTable 4-5.** The results of collinearity diagnosis in the linear regression (for selection of covariates in model 1 and model 2).


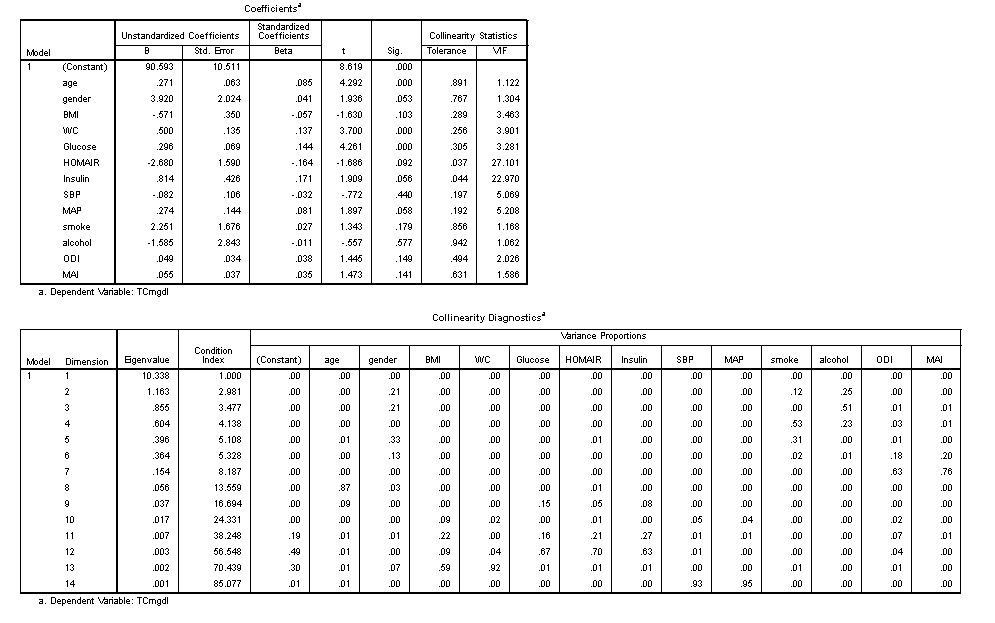


**sTable 6-7.** The results of collinearity diagnosis in the logistic regression (for selection of covariates in model 1 and model 2).


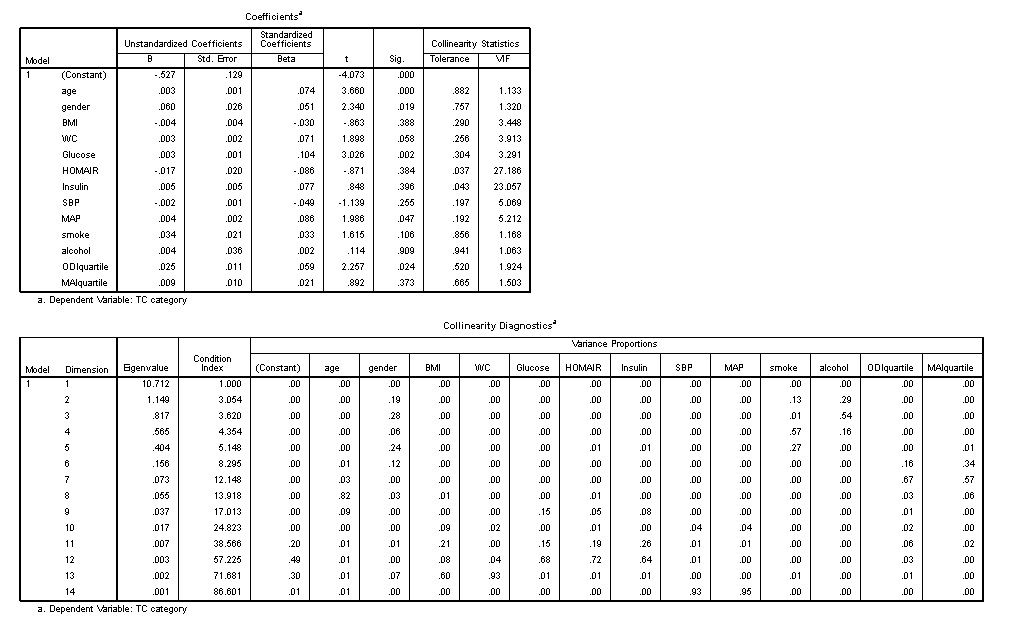


**sTable 8.** The results of Spearman’s correlations (for selection of covariates in model 3 and model 4).

**
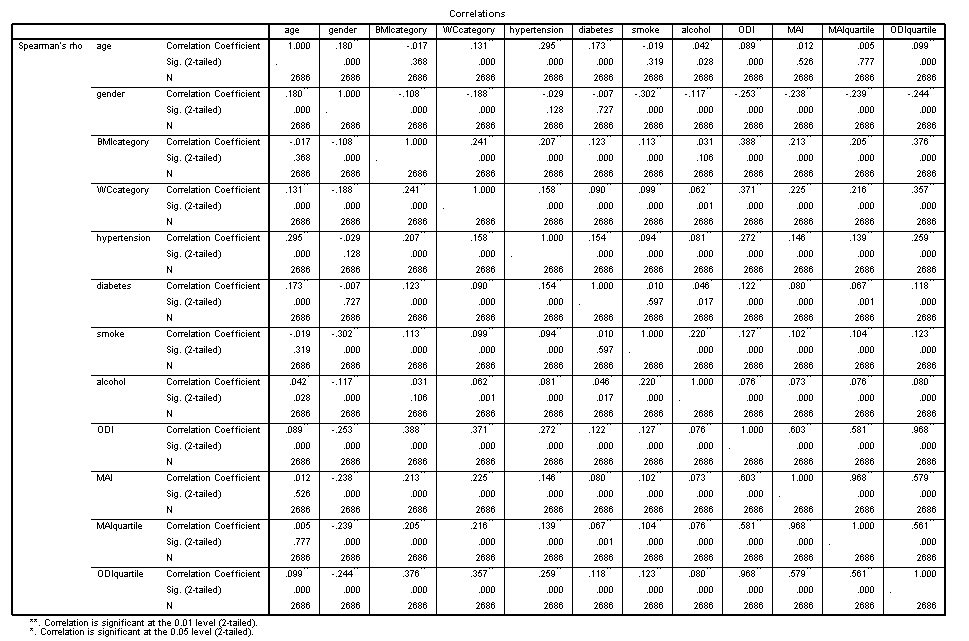
**

**sTable 9-10.** The results of collinearity diagnosis in the linear regression (for selection of covariates in model 3 and model 4).

**
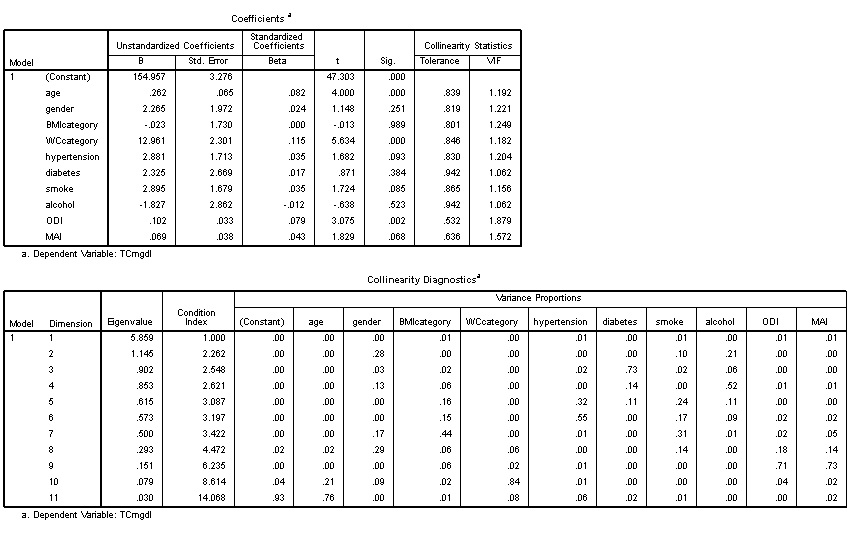
**

**sTable 11-12.** The results of collinearity diagnosis in the logistic regression (for selection of covariates in model 3 and model 4).

**
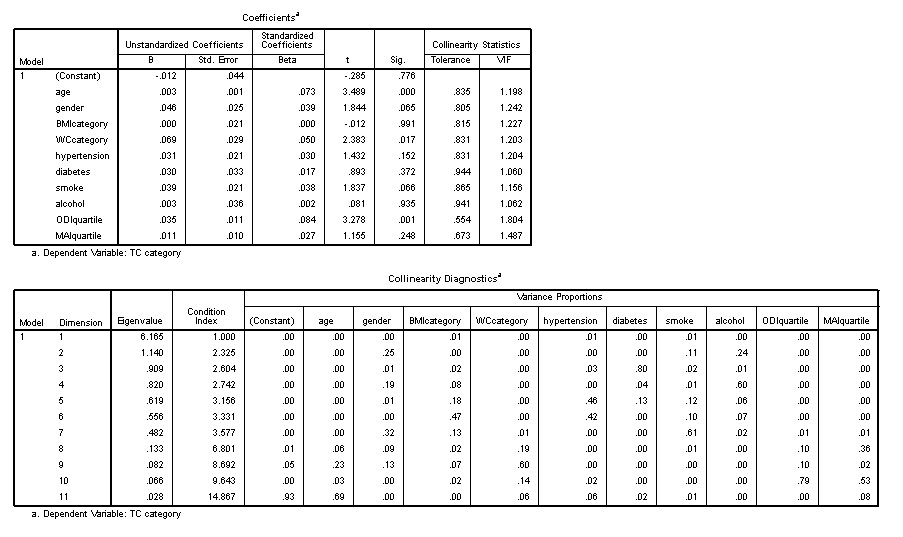
**

**sFigure 1, sFigure 2.**

**sFigure 1.**

**
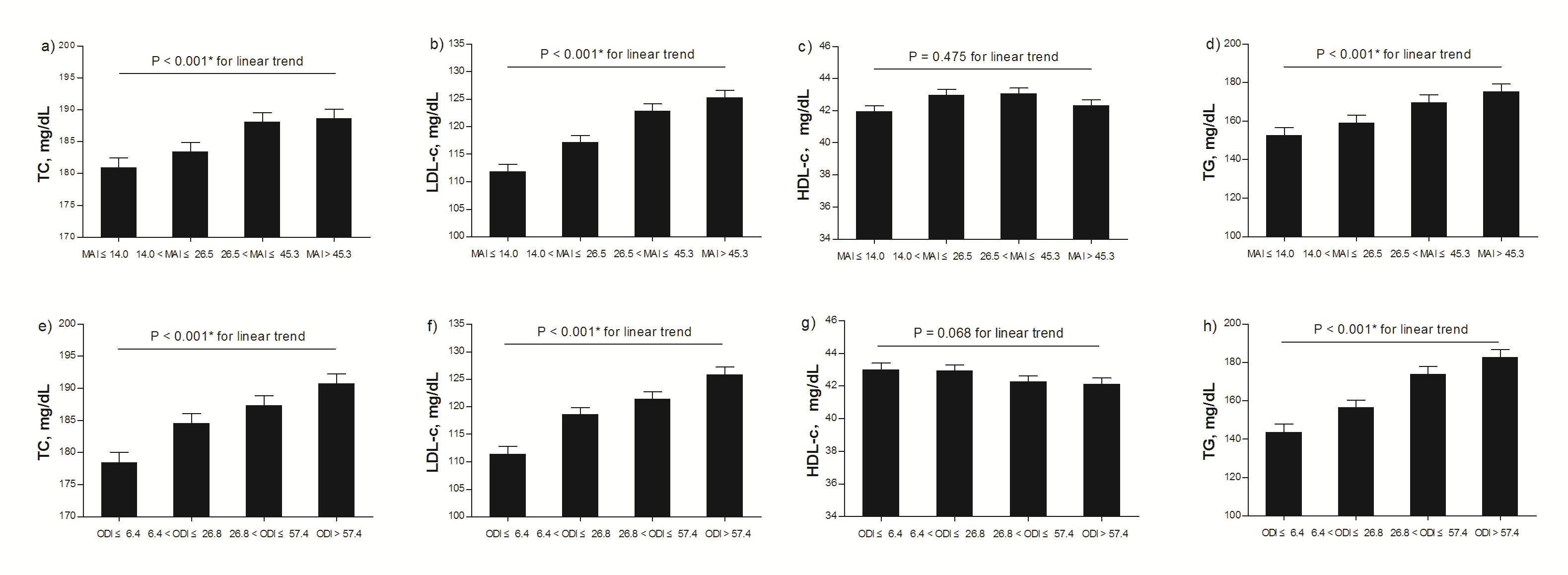
**

**sFigure 2.**

**
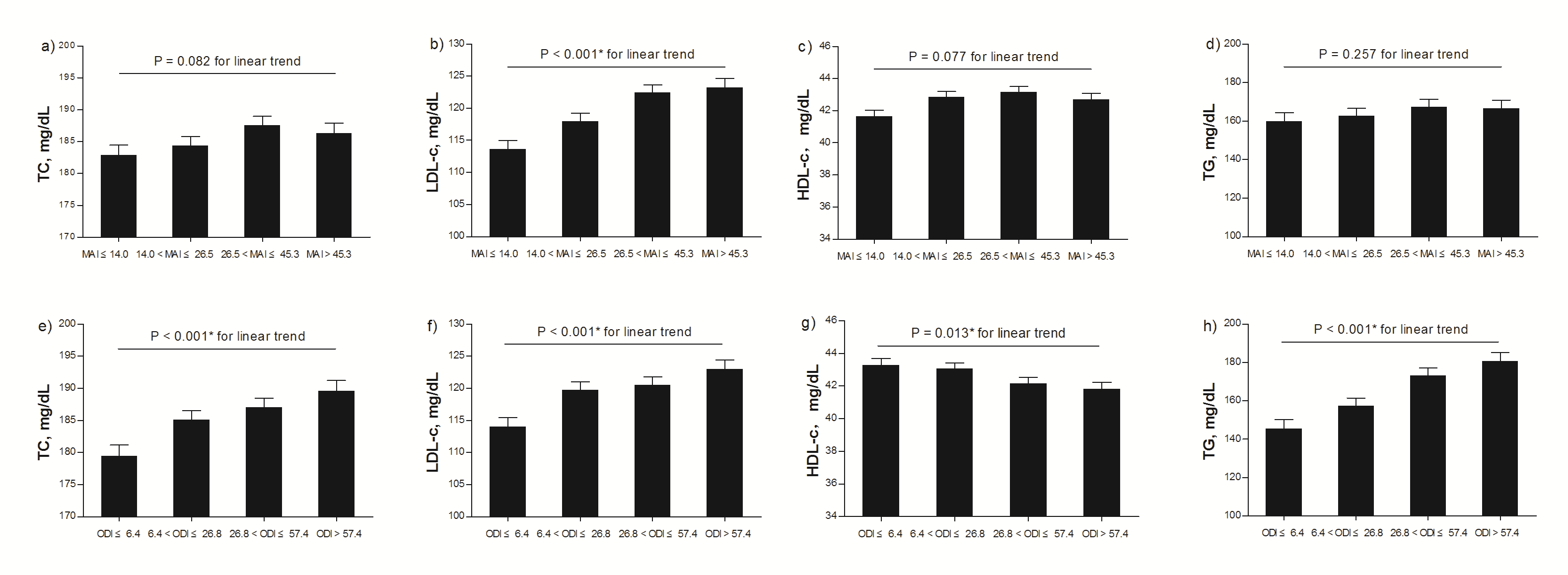
**
